# 在中国澳门吸烟人群中的肺癌筛查研究

**DOI:** 10.3779/j.issn.1009-3419.2021.101.25

**Published:** 2021-08-20

**Authors:** 晓战 张, 振荣 张, 鲲 程, 贞勇 杨, 伟国 朱, 伟文 周, 肇聪 林, 亚兵 曹, 牧 李

**Affiliations:** 1 中国澳门特别行政区，镜湖医院呼吸内科 Respiratory Medicine, Kiangwu Hospital, Macao, China; 2 中国澳门特别行政区，镜湖医院健康管理中心 Health Management Center, Kiangwu Hospital, Macao, China; 3 中国澳门特别行政区，镜湖医院影像科 Imaging Center, Kiangwu Hospital, Macao, China; 4 中国澳门特别行政区，镜湖医院胸外科 Thoracic Surgery, Kiangwu Hospital, Macao, China; 5 中国澳门特别行政区，镜湖医院肿瘤科 Oncology Department, Kiangwu Hospital, Macao, China

**Keywords:** 肺肿瘤, 筛查, 早期诊断, Lung neoplasms, Screening, Early diagnosis

## Abstract

**背景与目的:**

中国澳门肺癌发病率逐年上升，吸烟人群是肺癌的高发人群，本研究旨在了解中国澳门长期吸烟人群的肺癌发病情况及胸部低剂量计算机断层扫描(low-dose computed tomography, LDCT)肺结节特点。

**方法:**

通过澳门中华医学会会员私家医生推荐及宣传招募中国澳门无症状长期吸烟人士，行胸部LDCT检查，分析肺癌、肺部结节检出率及影像学特点。

**结果:**

符合纳入条件者291例，检出肺癌10例，检出率3.44%(95%CI: 2.78%-4.01%)，其中，肺腺癌5例，鳞癌、小细胞肺癌各2例，腺鳞癌1例。早期肺癌4例，占40%。212例检出肺结节，肺结节总检出率72.9%(95%CI: 67.8%-78.0%); 疑似肺癌结节44例，检出率15.1%(95%CI: 11.0%-19.2%)。单发结节51例，无肺癌检出; 多发结节161例，检出肺癌9例，两组肺癌检出率无统计学差异(*P* > 0.05)。 < 6 mm实性结节与 < 5 mm非实性结节组168例，未检出肺癌; ≥6 mm实性结节与≥5 mm非实性结节组44例，检出肺癌9例，两组比较有统计学差异(*P* < 0.05)。

**结论:**

长期吸烟人群中肺癌检出率高，类型以腺癌为主，肺部结节发生率高，当实性结节≥6 mm或非实性结节≥5 mm时，肺癌检出率增高。建议在符合高危因素的男性吸烟人群中推行胸部LDCT筛查肺癌，女性肺癌筛查，应重新界定高危因素。

## Abstract (en)

**Background and objective:**

Lung cancer incidence in Macao increases gradually, smoking is one of the important high risk factors. The purpose of this study is to observe the detection rate of lung cancer and nodules in long-term smoking Macao individuals.

**Methods:**

We recruited eligible Macao residents by publicity, all subjects were arranged to receive low-dose computed tomography screening. Image features of lung nodules were analyzed by radiologist. For suspicious lung cancer, multiple disciplinary team (MDT) was arranged.

**Results:**

A total of 291 were adopted, 10 lung cancers were detected, the detection rate of lung cancer was 3.44% (95%CI: 2.78%-4.01%), all were males. There were 5 adenocarcinoma patients, each 2 squamous-cell carcinoma and small cell lung carcinoma patients; 1 adenosquamous cancer patient. Among 10 lung cancers, 40% had stage 1 disease. The detection rate of lung nodules was 72.9% (95%CI: 67.8%-78.0%); The number of suspicious lung nodules were 44, and the detection rate was 15.1% (95%CI: 11.0%-19.2%). There was no significant differences in the lung cancer detection rate between the single and multiple lung nodule groups (*P* > 0.05). There were 168 subjects in the < 6 mm solid lung nodule (SN) and < 5 mm no-solid lung nodule (NSN) group and no lung cancer was found, 44 subjects in the ≥6 mm SN and ≥5 mm NSN group. All 9 lung cancer patients were detected in this group. The detection rate of lung cancer was higher than that of the < 6 mm SN and < 5 mm NSN group (*P* < 0.05).

**Conclusion:**

There are high detection rate of lung cancer and lung nodule in the long-term smoking individuals. The lung cancer rate increases when the lung nodule size is larger than 6 mm in SN and 5 mm in NSN. Adenocarcinoma is the major type in the smokers' lung cancers. We suggest long-term smokers should join in the future lung cancer screening trial in Macao. Female lung cancer screening should be established different standard.

世界卫生组织数据^[1]^显示，在亚洲地区肺癌发病率和死亡率逐年上升。中国澳门最新癌症登记年报^[2]^报道，肺癌仍是中国澳门癌症发病率和死亡率占首位的恶性肿瘤。研究发现，借助胸部低剂量计算机断层扫描(low-dose computed tomography, LDCT)可以检出直径 < 1 cm的小肺癌，早期肺癌检出率高达80%，可使肺癌死亡率减少20%^[3]^。本研究旨在研究中国澳门长期吸烟人群的肺癌及肺结节发病情况，为中国澳门开展大规模肺癌筛查提供参考。

## 1 对象与方法

### 1.1 研究对象

2017年5月-2019年12月通过澳门中华医学会会员私家医生推荐及报纸、互联网宣传，招募中国澳门无症状重度吸烟者，通过问卷筛出符合下列条件者，纳入研究组，进行胸部LDCT扫描。纳入标准：(1)50岁-75岁; (2)长期吸烟，吸烟指数在30包年及以上(吸烟的年数乘以每日吸烟的包数); (3)吸烟超过20包年，且符合以下条件之一者：①长期工作在密闭的有粉尘颗粒较多的环境，超过5年; ②有慢性阻塞性肺疾病(chronic obstructive lung disease, COPD)病史; ③有肺结核病史(已治愈); ④弥漫性肺纤维化病史; ⑤父母、兄弟姐妹中有恶性肿瘤病史或肺癌家族史; ⑥曾有鼻咽癌、乳腺癌、胸腺瘤等放疗病史(原发肿瘤稳定5年以上)。其中满足(1)+(2)两项，或(1)+(3)中①-⑥任意一项，纳入研究组。排除标准：(1)其他肺部疾病的急性期; (2)已有威胁寿命的健康问题; (3)有肺部手术史、近5年有癌症病史(皮肤癌、原位癌等除外)。

### 1.2 筛查流程

培训澳门中华医学会会员私家医生及镜湖医院门急诊医生，推荐长期吸烟人士，预约来镜湖医院健康中心填写“肺癌危险因素初筛表”。统一由事先完成专业培训的调查人员进行。健康中心医生介绍筛查的目的、意义以及参加筛查的获益和可能的风险，回答参与者的问题。参与者明白无误后在自愿的原则下签署知情同意书。医生同时进行吸烟史、肺癌家族史等肺癌高危因素的确认，并检查长期吸烟体征，如牙齿、手指烟渍等，行呼出气一氧化碳浓度测试。符合条件者，填写“肺癌危险因素确认表”。并开具胸部LDCT检查单及肺功能检查单。

### 1.3 LDCT方法

按照中华医学会放射学会标准^[4]^。扫描参数：120 kVp; 45 mAs; 螺距0.938;重建层厚1.25 mm连续(层间隔为0)，扫描层厚1.25 mm。扫描范围：从肺尖到肋膈角(包括全部肺)，患者吸气末一次屏气完成扫描，扫描采样时间≤10 s，CT扫描探测器16排，不需要造影剂。图像存储：将1.25 mm层厚连续图像重建图像传入PACS。图像观察：由有经验的主治医师以上放射科医师在CT工作站观察图像，采用标准肺窗、纵隔窗(软组织窗)观察图像，肺窗的窗宽为1, 600 HU，窗位为-600 HU。结节测量：用电子测量尺通过结节最大截面测量其长径及宽径。说明：长径指最大截面的最大径; 宽径指与长径垂直的最大径。实性结节包括部分实性结节，直径测量包括非实性部分。所有≥2 mm的实性结节、非实性结节均在报告中描述。≥6 mm实性结节及≥5 mm非实性结节为肺癌可疑结节。

### 1.4 随诊

首次胸部LDCT检查发现肺部可疑恶性结节者，经肺癌筛查小组医生讨论后根据肺癌危险程度及LDCT影像特点安排正电子发射型计算机断层显像(positron emission computed tomography, PET)、胸部CT复查、支气管镜下肺活检、超声内镜引导下的经支气管针吸活检(endobronchial ultrasound-guided transbronchial needle aspiration, EBUS-TBNA)、或胸腔镜下肺切除术，明确诊断及行下一步治疗。流程按照周清华等推荐的“中国肺结节分类、诊断与治疗指南” ^[5]^中图 3(初次/基线CT扫描)进行。随访：对所有研究对象自行LDCT检查日起由肺癌筛查小组随访2年。主要随访内容有吸烟状况、是否戒烟、高危因素的防护、呼吸系统疾病的症状、再次检查时间。确诊肺癌者：主要随访内容有治疗方案、生存期。

### 1.5 统计学方法

采用SPSS 20.0软件进行统计分析。计量资料以均数±标准差(Mean±SD)表示，肺癌大小及多发结节组与单发结节组肺癌检出率比较均采用卡方检验，相关分析采用*Pearson*双变量相关分析，*P* < 0.05认为差异具有统计学意义。

## 2 结果

### 2.1 一般资料及肺癌高危因素情况

符合条件并完成胸部LDCT检查者291例，平均年龄(60.23±5.53)岁(50岁-75岁)。性别比例、年龄分布、教育程度、吸烟指数及体征、家族肺癌和肿瘤史、工作粉尘接触史、肺部陈旧疾病等情况见表 1。其中有戒烟愿望74例。87例有家族肿瘤病史中，父亲有43例，母亲有17例，兄弟姐妹有17例，家族中2人以上者有5例，其他5例。

**表 1:** 研究对象一般特点 Characteristics of participants

Items		Characteristic	Data [*n* (%)]	LC (*n*)
Gender		Male	282 (96.9)	10
		Female	9 (3.1)	0
Age groups (yr)		50-54	48 (16.5)	0
		55-59	87 (29.9)	5
		60-64	90 (30.9)	2
		65-69	49 (16.8)	2
		70-75	17 (5.8)	1
Education level		No	12 (4.1)	2
		Primary school	94 (32.3)	4
		Junior high school	99 (34.0)	1
		High school	81 (27.8)	3
		Tertiary and college	5 (1.7)	0
Profession		Dust	91	6
		No-dust	200	4
		Harm	211	2
		No-harm	90	8
Smoking	PACK-years	≥30	289 (99.3)	9
		20-29	2 (0.7)	1
	Duration (yr)	15-19	2 (0.7)	0
		20-29	19 (6.5)	1
		≥30	270 (92.8)	9
	Pack	0.5-1	131 (45.0)	2
		1-1.5	123 (42.3)	6
		> 1.5	37 (12.7)	2
		Family member smoking	236 (81.1)	9
		Room member smoking	156 (53.6)	4
		Smoke spot in fingers	263 (90.4)	10
		Smoke spot in teeth	264 (90.7)	10
Family lung cancer history		Yes	24 (8.2)	1
		No	267 (91.8)	9
Other cancer family history		Yes	87 (29.9)	1
		No	204 (70.1)	9
Lung disease history		Yes	92 (31.6)	6
		No	199 (68.4)	4
Any high risk occupation		Yes	215 (73.9)	10
		No	76 (26.1)	0
LC: lung cancer.				

### 2.2 肺结节特点

212例检出肺部结节，79例未检出，检出率72.9%(95%CI: 67.8%-78.0%)。结节大小及分布特点见表 2和图 1。表 2和图 1中可以看出，< 6 mm多发实性结节检出率最高，其次为 < 6 mm单发实性结节。可疑肺癌结节44例，为≥6 mm实性结节和≥5 mm非实性结节，检出率15.1%(95%CI: 11.0%-19.20%)，可疑肺癌结节组肺癌检出率高于 < 6 mm实性结节与 < 5 mm非实性结节组(*χ*^2^=31.0, *P* < 0.05)，具体见表 4。进一步相关分析发现，结节大小与肺癌检出率相关，*r*=0.43，*P* < 0.01。单发结节51例，无肺癌检出; 多发结节161例，检出肺癌9例，两组肺癌检出率无统计学差异(*χ*^2^=1.76, *P* > 0.05)，具体见表 5，进一步相关分析发现，结节数目与肺癌检出率无相关性，*r*=0.09，*P* > 0.05。结节随访见图 2。

**表 2:** 肺结节大小分布 Lung nodule size distribution in participants

Item	Solid lung nodules (*n*)				Ground glass nodules (*n*)		
	< 6 mm	6 mm-8 mm	> 8 mm		< 5 mm	5 mm-10 mm	> 10 mm
No.	92	275	273		267	285	285
Single	58	15	17		15	5	4
≥2	141	1	1		9	1	2
Total	291	291	291		291	291	291
No.: The number of participants who were no lung nodules.							

**图 1:**
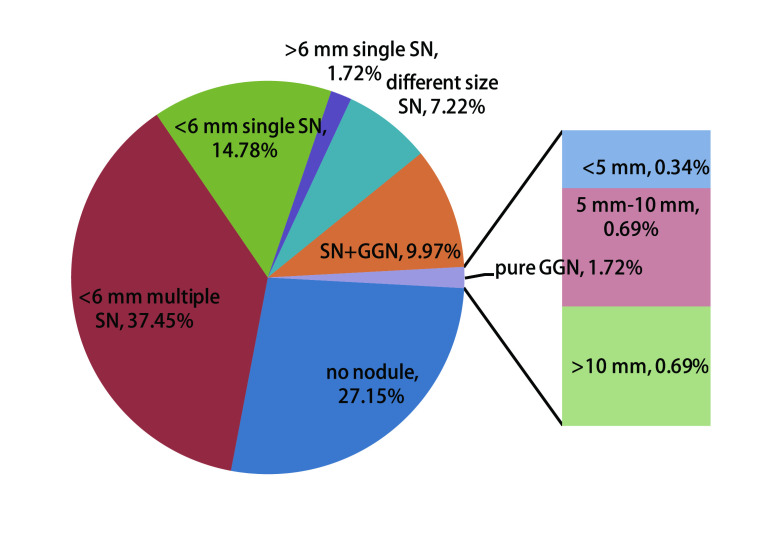
结节分布百分比图 Percentage distribution graph of lung nodules. SN: solid nodule; GGN: ground glass nodule.

**表 3:** 212例检出肺结节者分布情况 Lung nodule distribution in 212 participants

Item	Single (*n*)	≥2 (*n*)	GGN (*n*)			Total [*n* (%)]
			< 5 mm	5 mm-10 mm	> 10 mm	
< 6 mm SN	43	109	13	3	4 (2)	172 (59.1)
6 mm-8 mm SN	3	9	4	0	0	16 (5.5)
> 8 mm SN	2	12 (5)^*^	2 (1)^*^	1	2 (1)^*^	19 (6.5)
< 5 mm GGN	1	2	/	/	/	3 (1.0)
5 mm-10 mm GGN	2	0	/	/	/	2 (0.7)
> 10 mm GGN	0	0	/	/	/	0 (0)
Total [*n* (%)]	151 (17.5%)	132 (45.4%)	19 (6.5%)	4 (1.4%)	6 (2.1%)	212 (72.9%)
() ^*^: numbers of lung cancer patients; SN: solid nodule; GGN: ground glass nodule.						

**表 4:** 不同大小结节组肺癌检出率比较 Comparison of the detection rate of lung cancer among different size nodule groups

Groups	LC (*n*)	No-LC (*n*)	Total	Detection rate (%)
A group (< 6 mm SN + < 5 mm GGN)	0	168	168	0
B group (≥6 mm SN + ≥5 mm GGN)	9	35	44	20.5
Total	9	203	212	4.25
No-LC: no lung cancer. There was statistical differences between two groups (adjust *χ*^2^=31.03, *P* < 0.01);The detection rate of lung cancer in B group was higher than A group. SN: solid nodule; GGN: ground glass nodule.				

**表 5:** 单发与多发结节组肺癌检出率比较 Comparison of the detection rate of lung cancer between single and multiple nodule groups

Groups	Lung caner (*n*)	No-lung cancer (*n*)	Total	Detection rate (%)
Single group	0	51	51	0
Multiple group	9	152	161	5.59^*^
Total	9	203	212	4.25
Single group: single nodule group; Multiple group: multiple nodule group. ^*^: There was no statistical differences between single and multiple nodule groups (*χ*^2^=1.76, *P* > 0.05).				

**图 2:**
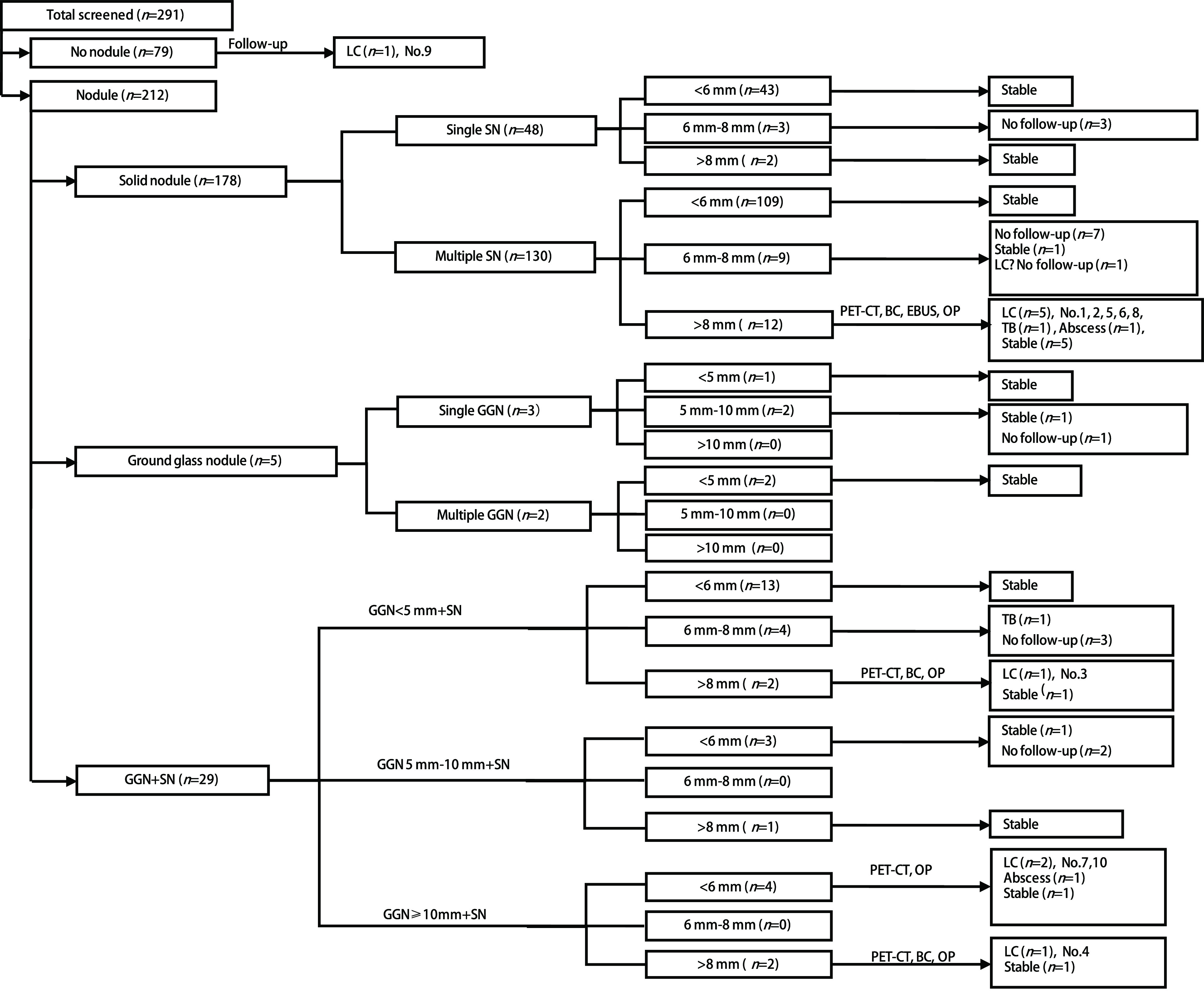
肺癌LDCT筛查结节随诊图 Management algorithm for pulmonary nodules detected by LDCT scans. LDCT: low-dose computed tomography; LC: lung cancer; BC: bronchoscopy; EBUS: endobronchial ultrasound; OP: operation; SN: solid nodule; GGN: ground glass nodule; n: number. No.: the lung cancer patients code which corresponds with the Table 6.

### 2.3 肺癌患者诊断及治疗

共检出10例肺癌患者，均为病理证实，检出率3.44%(95%CI: 2.78%-4.01%)。早期肺癌(TNM分期I期)4例，占肺癌病例40%。腺癌5例，占50%。其中表 6中第9例患者，首次行LDCT未见结节，21个月后因“咳嗽1月”门诊就诊，胸片发现肿块，行胸CT及支气管镜考虑肺癌、行化疗后放疗，2020年12月复诊，发现肿瘤进展。余9例均为首次LDCT疑为肺癌。1例IIIa期肺腺癌，行同步放化疗，生存19个月。2例(small cell lung cancer, SCLC)广泛期行化疗，分别生存10个月、14个月。2例PET-CT检查高度怀疑肺癌患者，不同意支气管镜等检查，随诊无增大，未计入肺癌组。具体见表 6。

**表 6:** 肺癌患者特征、分期、治疗及随访 Characteristics, stage, treatment and follow-up of lung cancer patients

No.	Age (yr)	Location	Size (mm)	Histology	TNM	Stage	Gene mutation	Treatment	Follow-up
1	68	RUL	20×15 SN	Ade.	T1bN2M0	cIIIa	*EGFR* (-)	Chemoradiation	19 mon
							*ALK* (-)		
2	64	LUL	33×19 SN	Ade.	T2aN1M0	pIIb	*EGFR* (+)	Surgery	No
							*ALK* (-)		
3	58	Left hilum	39×44 SN	SCLC		ext.	No	Chemoradiation	10 mon
4	59	LUL	31×20 SN	Ade+Squa.	T2aN1M0	pIIb	*EGFR* (+)	Surgery	Dete.
							*ALK* (-)		
5	59	Left hilum	62×48 SN	SCLC		ext.	No	Chemoradiation	14 mon
6	64	RUL	9×10 SN	Squa.	T1aN0	pIa1	No	Surgery	Stable
7	56	RLL	11×12 GGN	Ade.	T1bN0	pIa1	*EGFR* (-)	Surgery	Stable
							*ALK* (-)		
8	74	LUL	23×26 SN	Ade.	T1cN0	pIa3	EGFR (-)	Surgery	Stable
							*ALK* (-)		
9	59	LLL	19×18 SN	Ade.	cT1bN2M1	cIIIa	*EGFR* (-)	Chemoradiation	Dete.
							*ALK* (-)		
10	65	RLL	13×17 GGN	Squa.	T1bN0	pIa3	No	Surgery	Stable
RUL: right upper lobe; LUL: left upper lobe; RLL: right lower lobe; LLL: left lower lobe; Ade.: adenocarcinoma; Squa.: squamous cancer; Ade+Squa.: adenosquamous cancer; ext.: extensive stage; Dete.: deterioration; TNM: tumor-node-metastasis; EGFR: epidermal growth factor receptor; ALK: anaplastic lymphoma kinase; SN: solid nodule; GGN: ground glass nodule.									

10例肺癌患者，8例为实性结节(包括表 6第9例患者)，直径均 > 8 mm，其中7例合并 < 6 mm实性结节。2例毛玻璃结节，直径均 > 10 mm，亦合并 < 6 mm实性结节。其中1例腺鳞癌患者(表 6第4例患者)，LDCT发现直径均 > 10 mm GGO 5个，< 6 mm实性结节及 < 5 mm非实性结节多个，并左上支气管狭窄，行支气管镜活检病理为鳞癌，PET-CT检查发现左肺门实性结节有摄取，右肺结节无摄取。后行左肺全切，病理诊断为腺鳞癌，术后行放化疗。行左肺全切后病理示腺鳞癌，肺内小结节为原位腺癌，1枚淋巴结见鳞癌转移，计为实性结节组。典型LDCT图像见图 3。

**图 3:**
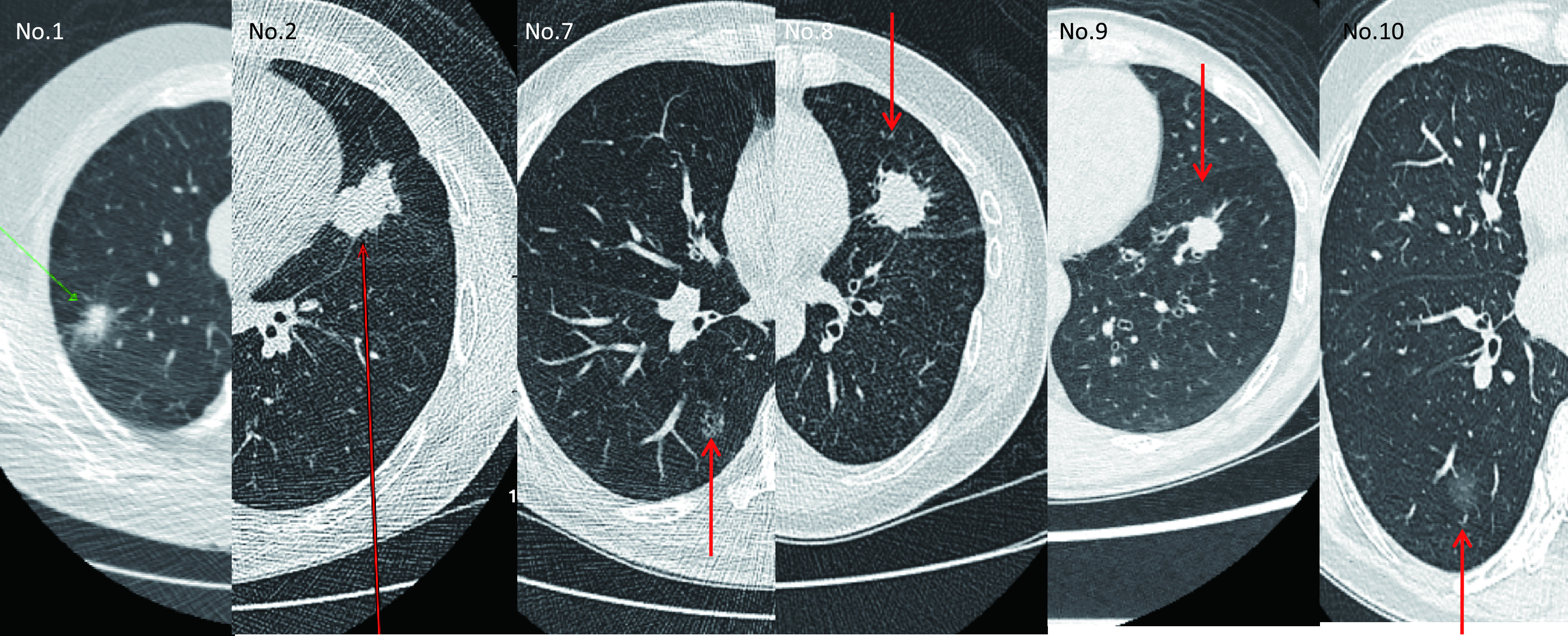
部分肺癌患者CT图。^*^No.：和表 6对应的肺癌患者编号。箭头分别指部分实性结节、实性结节及毛玻璃结节。 CT images of lung cancer patients. ^*^No.: The lung cancer patients'code which corresponds with the Table 6. Arrows point to semi-SN, SN and GGN.

### 2.4 吸烟与肺部结节、肺癌的相关性分析

291例入组者手指烟渍、牙齿烟渍分为3级，为明显烟渍、轻度烟渍和未见烟渍; 分别和每日吸烟量、烟龄、实性结节数、结节大小、肺癌行相关性分析。结果显示手指烟渍与烟龄(*r*=0.21, *P* < 0.05)、实性结节数(*r*=0.13, *P* < 0.05)呈正相关; 牙齿烟渍与烟龄呈正相关(*r*=0.24, *P* < 0.05)，余无相关性。

## 3 讨论

2018年中国澳门新发肺癌351例，50岁以上男性213例，粗发病率53.3/10万人口^[2]^。本组检出肺癌10例，均为男性，检出率为3.44%，高于同地区发病率，也高于周清华等^[6]^在国内7个省市的多中心筛查的肺癌检出率0.1%-2.9%，中国台湾^[7]^的1.7%和美国^[3]^的1%、韩国^[8, 9]^、日本^[10]^的0.36%-1.1%。分析可能的原因为：第一，吸烟史可靠。为避免不符合条件人士入组，设置吸烟相关问题、检查吸烟相关体征及呼出气一氧化碳浓度测试确认。首诊医生查体发现手指烟渍及牙齿烟渍分别高达90.4%和90.7%，而手指烟渍、牙齿烟渍与烟龄均呈正相关。同类研究未见吸烟体征等描述，易混杂吸烟量不足人士入组致肺癌检出率减低。第二，合并其他肺癌高危因素高于同类研究。其中，53.6%有成年后二手烟接触史; 81.1%有童年时期家庭成员吸烟史。73.9%从事建筑等接触粉尘职业，31.6%既往有肺结核或合并COPD等肺部慢性疾病史。第三，男性占96.9%，而男性肺癌发病率高于女性。本组研究对象多居住于中国澳门人口密度最高的黑沙环、筷子基区，居住空间狭小，空气污染也可能是肺癌检出率高的原因之一。国内流行病学调查^[11]^发现大气污染指数与肺癌发病率密切相关。早期肺癌检出率40%，低于国内外基线水平^[12]^。与本组患者文化程度低、对肺癌防护意识差有关。本组检出肺癌患者年龄在58岁-74岁之间，若这类肺癌高危因素人士，按照国内指引^[13]^，50岁后每年行LDCT检查，则可早诊早治，提高肺癌治愈率。因此，应加大对吸烟人群的健康教育，提高肺癌筛查参与度。

有研究^[14-16]^发现，肺腺癌在亚洲非吸烟女性中发病率高。本研究5例肺腺癌患者均为男性，男性肺腺癌检出率占50%，高于与吸烟相关的鳞癌和小细胞肺癌。可能与中国澳门烟草市场多为进口过滤卷烟有关。过滤卷烟使烟草烟雾成分发生了改变，过滤香烟的使用理论上可减少尼古丁、焦油和一氧化碳的含量，但由于吸烟者的补偿行为如堵住滤嘴上透气孔、加大吸入烟草烟雾量等，并没有减少吸烟者体内的尼古丁和焦油含量，而加大烟草烟雾量和过滤嘴的使用，使小分子烟雾更易到达外周气道，造成腺癌高发^[17, 18]^。另外，助燃剂的使用使一氧化氮增加，促进亚硝胺类物质的形成，而亚硝胺-4-(甲基化亚硝胺类)-1-(3-吡啶基)-1-丁酮与肺腺癌密切相关^[19]^。PM2.5是空气污染物中的重要组成成分，可刺激肺部细胞一氧化氮的表达，也可能是原因之一。亚洲女性肺腺癌与表皮生长因子受体(epidermal growth factor receptor, *EGFR*)基因变异有关，占到50%以上^[14-16, 20]^。本组男性肺癌只有1例*EGFR*基因变异，占20%，低于女性。推测与男性腺癌基因位点的不同变异有关，还需进一步探讨。中国医学科学院肿瘤医院对2000年-2012年诊治的男性肺癌进行趋势研究发现，吸烟者肺腺癌从15.25%上升至41.85%，每年以6.8%的水平递增^[21]^。因此，男性吸烟肺腺癌的发病机理值得关注研究。肺结节检出率国内外报导差异较大，美国Swensen^[22]^对1, 502例参与者的结节检出率为51.3%。英国的UKLS项目^[23]^中，1, 994名参与者，发现肺结节率50.9%。上海^[24]^对某社区2, 972名60岁-75岁的老人行LDCT检查，吸烟组的结节检出率33.7%。本研究肺结节检出率高达72.9%，除与以上吸烟、粉尘等因素有关外，也与本研究的扫描层厚1.25 mm，重建层厚1.25 mm连续(层间隔为0)，可发现2 mm及以上的微小结节有关。研究^[25]^发现，< 6 mm实性结节及 < 5 mm非实性结节肺癌发生率低，肺癌危险性 < 1%，在NLST中被划为假阳性结节。随着结节增大，肺癌危险度增高。本研究与此结果类似。即可疑结节组肺癌检出率明显高于假阳性结节组。说明结节大小与肺癌危险性相关。也与Larici等^[26]^的结果一致。

肺结节以多发实性结节为主，占74.6%，高于NELSON^[27]^研究发现的49.5%。多发肺结节组肺癌检出率有增高趋势，但无统计学差异。与NELSON^[27]^研究类似，即结节多少与肺癌发生率无明显相关。在多发结节中，NELSON研究指出97%的肺癌发生在最大结节。本组9例肺癌患者，均为多发结节，术后病理证实肺癌均在最大结节。近期研究发现，在高危人群中，即使假阳性结节，在随后的4年-10年间，肺癌发生率也明显高于低危人群，特别是同一肺段^[28]^出现新发结节时，尤其应该引起注意。本组表 5第9例肺癌患者，即为新发结节。因此，对于此类高危人群，无论有无结节，无论结节大小，50岁后均应每年一次LDCT检查，才能早期发现肺癌。但LDCT检查，有放射风险，如何控制风险，提高肺癌检出率，仍需不断研究出更敏感、特异度更高的筛查技术。

本组女性占3.1%，共9例，均未发现肺癌。与中国澳门女性吸烟者少，能够达到20包年及以上烟量的女性更少有关。中国澳门控烟评估报告中，2018年女性45岁以上吸烟率仅为2.4%，不到男性吸烟的10%。而2018年中国澳门女性肺癌新发128例，50岁以上新发117例，是男性肺癌的50%(男性50岁以上213例)^[2]^。因此，应用以吸烟为主的纳入条件会使女性肺癌漏检，建议重新界定女性肺癌高危因素，如年龄50岁以上、家族肺癌病史、二手烟接触史、工作环境粉尘接触史等。

综上所述，吸烟人群中LDCT以多发实性小结节为主，肺癌类型以腺癌为主，当实性结节≥6 mm或非实性结节≥5 mm时，肺癌发病率增高。建议在符合高危因素的男性吸烟人群中推行胸部LDCT筛查肺癌，女性肺癌筛查，应重新界定高危因素。
